# A guide for establishing patient-derived organoids from bile samples obtained during endoscopic procedures and performing gene expression knockdown

**DOI:** 10.3389/fcell.2026.1812445

**Published:** 2026-05-29

**Authors:** Carla Rojo, Juan J. Vila, Laura Guembe, Amaia Arrubla-Gamboa, Vanesa Jusué-Irurita, Juan Carrascosa-Gil, María Rullan, Javier Rández-Garbayo, Maite G. Fernández-Barrena, Meritxell Huch, Jesús Urman, Matías A. Ávila, Carmen Berasain, Maria Arechederra

**Affiliations:** 1 Hepatology Laboratory, Solid Tumors Program, CIMA, CCUN, University of Navarra, Pamplona, Spain; 2 Department of Gastroenterology and Hepatology, Navarra University Hospital, Pamplona, Spain; 3 IdiSNA, Navarra Institute for Health Research, Pamplona, Spain; 4 Morphology Core Facility, Center for Applied Medical Research (CIMA), University of Navarra, Pamplona, Spain; 5 National Institute for the Study of Liver and Gastrointestinal Diseases (CIBERehd, Carlos III Health Institute), Madrid, Spain; 6 Max Planck Institute of Molecular Cell Biology and Genetics, Dresden, Germany; 7 Center for Systems Biology (CSBD), Dresden, Germany

**Keywords:** 3D cultures, bile duct, biliary models, biliary stenosis, cholangiocarcinoma, cholangiocytes, organoid gene knockdown

## Abstract

Bile represents a clinically accessible biological fluid that can mitigate major limitations associated with tissue-based sampling for the generation of organoid models to study hepatobiliary disease, including biliary tract cancers, where tissue availability is often limited. Importantly, bile can also enable the generation of non-malignant cholangiocyte organoids that are otherwise difficult to obtain. Here, we describe an operator-oriented, step-by-step protocol to generate organoids from fresh bile collected during endoscopic retrograde cholangiopancreatography (ERCP), together with two complementary workflows for siRNA delivery in 3D cultures. We detail critical control points that are often under-reported, yet considerably influence success and reproducibility. The protocol was optimized and applied in a real-world cohort of 21 patients undergoing ERCP, including benign biliary obstruction due to choledocholithiasis (n = 5) and malignant strictures (n = 16: cholangiocarcinoma; n = 13: gallbladder adenocarcinoma; n = 1: ampullary tumors; n = 2). Expandable organoids were established in 17/21 cases (81%), with establishment rates of 60% for choledocholithiasis and 85%–100% across malignant entities. The anticipated results include organoid outgrowth within ∼2–3 weeks and morphological heterogeneity in cultures derived from malignant strictures, where normal-like and tumor-like populations may initially coexist and can drift toward a cystic phenotype under routine expansion, motivating optional manual handpicking when tumor-enriched lines are required. As downstream readouts, we show the feasibility of DNA-based profiling in selected paired bile–organoid samples (targeted sequencing and ULP-WGS copy-number analysis) and demonstrate proof-of-concept gene silencing via siRNA in both dissociated cells prior to re-embedding and intact fully formed organoids while preserving the 3D architecture. Collectively, this workflow provides a practical and reproducible framework to establish, expand, characterize, and functionally perturb bile-derived organoids from routine clinical procedures, facilitating standardized implementation across laboratories.

## 1 Introduction

Organoids are a versatile *in vitro* platform with broad applications ranging from pathophysiology studies to drug discovery and toxicity testing (Method of the Year, 2017: Organoids, 2018; Corrò et al., 2020; Driehuis et al., 2020). Although the outcomes may vary across laboratories due to differences in the source material, culture conditions, and operator-dependent steps, patient-derived organoids have consistently been shown to retain key genetic and phenotypic features of the original tissue and provide reproducible, clinically informative experimental models (van de Wetering et al., 2015; Clevers, 2016; Hu et al., 2018; Sachs et al., 2018; Driehuis et al., 2020).

Biliary tract cancers (BTCs), encompassing cholangiocarcinoma (CCA) and gallbladder carcinoma (GBC), are highly lethal malignancies characterized by late diagnosis and limited therapeutic options (Benavides et al., 2015; Banales et al., 2020; Okumura et al., 2021). A major hurdle in BTC research and precision medicine is the scarcity of robust preclinical models that faithfully recapitulate a patient’s tumor biology (Amato et al., 2020; Krendl et al., 2025).

Liver organoids can be generated from different sources such as tissue biopsies and pluripotent stem cells (Huch et al., 2013; Takebe et al., 2013; Broutier et al., 2017; Nuciforo and Heim, 2021; Bregante et al., 2026). However, access to the biliary epithelium is often limited, and obtaining nonmalignant cholangiocytes as controls is particularly challenging due to the invasiveness of sampling and the suboptimal quality of advanced disease-stage tissues. In this context, bile represents an accessible biospecimen and a practical starting material for biliary organoid derivation (Soroka et al., 2019b; Roos et al., 2021b; Kinoshita et al., 2023). In this context, our group established the BileBank project (https://bilebank.org/) as an international initiative to promote and advance the use of bile collected during endoscopic retrograde cholangiopancreatography (ERCP) as a liquid biopsy matrix. ERCP is routinely performed in tertiary care referral hospitals for both diagnostic evaluation and therapeutic management of biliary strictures originating from benign conditions such as choledocholithiasis and from biliary tract and pancreatic malignancies.

As previously demonstrated, bile collected during endoscopic procedures without representing any additional risk for the patients can be leveraged as a liquid biopsy matrix. For instance, we showed that targeted mutational analysis of bile cell-free DNA can discriminate benign from malignant biliary strictures, supporting early cancer detection (Arechederra et al., 2022; 2025). Moreover, we have shown that protein- and lipid-related signatures (Urman et al., 2020), cfDNA methylation biomarkers (Loi et al., 2022), and microbially amidated bile acids (Temprano et al., 2025) can provide complementary clinically relevant information (Arechederra et al., 2024).

Building on the availability of fresh bile samples across benign and malignant biliary diseases, we have optimized a robust workflow to establish bile-derived organoids. In addition, to enable functional interrogation beyond descriptive characterization, we implement an optimized approach for siRNA delivery into 3D cultures, both disaggregated and fully formed, facilitating gene-silencing studies in bile-derived organoids.

## 2 Materials and equipment

### 2.1 Study design

Bile samples were collected from a cohort of 21 individuals with biliary strictures, who were scheduled to undergo ERCP at Navarra University Hospital (Pamplona, Spain) between January 2023 and December 2025. The demographic and clinical characteristics are summarized in Table 1. The study was approved by the Research Ethics Committee of the Navarra University Hospital (Pamplona, Spain; protocol number CEIm PI_2016/91). All the participants were aged ≥18 years and provided written informed consent for sample collection, analysis, and use of clinical data.

**TABLE 1:** Characteristics of patients with biliary strictures.

Characteristics		N°	Percentage
Gender, n (%)	Female	11	(52.4%)
	Male	10	(47.6%)
Median age years, n (range)	​	75	(50-92)
Location of biliary strictures, n (%)	Distal	8	(38.1%)
	Perihilar	8	(38.1%)
	nd	5	(23.8%)
CA19–9, U/mL n (%)	<500	11	(52.4%)
	≥500	6	(28.6%)
	nd	4	(19.0%)

Values are expressed as median (range) or n (%). Clinical data correspond to patients with biliary strictures undergoing ERCP for bile sampling and organoid derivation. CA19-9 levels were dichotomized according to a clinically relevant cut-off (500 U/mL). Abbreviations: CA19-9, carbohydrate antigen 19-9; ERCP, endoscopic retrograde cholangiopancreatography; nd, not determined.

### 2.2 Human bile samples: collection and transport

Bile was collected from overnight-fasted participants during standard ERCP performed by experienced endoscopists, as previously described (Urman et al., 2020). Briefly, after cannulation of the bile duct and prior to contrast injection, bile was aspirated through the sphincterotome into sterile additive-free tubes. Usually, 4 mL–15 mL of bile is collected. Samples were immediately transported to the laboratory at 4 °C (on ice) and processed within 2 h of collection to initiate the organoid establishment protocol.

NOTE#1. Longer preprocessing times were not evaluated in this study.

### 2.3 Reagents and consumables

Reagents for erythrocyte lysis: ACK buffer (ammonium chloride–potassium bicarbonate–EDTA) (prepared in-house; see Section 2.5).

- Ammonium chloride (NH_4_Cl) (Sigma-Aldrich; cat. no. A9434)
- Potassium bicarbonate (KHCO_3_) (Sigma-Aldrich; cat. no. 60339)
- Disodium EDTA (Sigma-Aldrich; cat. no. E9884)

Reagents for organoid culture, passaging, and cryopreservation.

- Advanced DMEM/F12 (Gibco; cat. no. 12634-010)
- Glutamine/penicillin/streptomycin (100×) (Gibco; cat. no. 10378-016)
- HEPES solution 1 M (Sigma; cat. no. H3537-100 ML)
- 3dGRO™ L-WNT Conditioned Media Supplement (Merck Millipore; cat. no. SCM105)
- Primocin, 500x (InvivoGen; cat. no. ant-pm-2)
- B27 supplement, 50x, minus vitamin A (Gibco; cat. no. 12587-010)
- N2 supplement, 100x (Gibco; cat. no. 17502-048)
- Nicotinamide (Merck; cat. no. N0636-100G)
- N-Acetylcysteine (NAC) (Sigma-Aldrich; cat. no. A8199-10G)
- Y-inhibitor (Tocris Bioscience; cat. no. Y-27632)
- [Leu15]-Gastrin I human (Sigma-Aldrich; cat. no. G9145-.1 MG)
- Human HGF (PeproTech; cat. no. 100-39H)
- Human FGF10 (PeproTech; cat. no. 100-26)
- Human EGF (PeproTech; cat. no. AF-100-15)
- A83-10 (TGFb inhibitor) (Merck; cat. no. SML0788)
- Forskolin (Calbiochem; cat. no. 344270)
- Dulbecco’s phosphate-buffered saline (PBS) (Gibco; cat. no. 14190-094)
- Dimethyl sulfoxide (DMSO) (Sigma-Aldrich; cat. no. D2650)
- Bovine serum albumin (BSA) (Sigma-Aldrich; cat. no. A9647)
- TrypLE Express Enzyme (Gibco; cat. no. 11598846)
- Recovery™ Cell Culture Freezing Medium (Gibco; cat. no. 12648-010)
- Matrigel Basement Membrane Matrix, LDEV-free, 10 mL (Corning; cat. no. 354234)

#### 2.3.1 Critical handling

1) Aliquots upon receipt were stored at −20 °C (or according to the manufacturer’s recommendations) to avoid repeated freeze–thaw cycles. 2) Thawing was done on ice or at 4 °C (e.g., overnight in a 4 °C refrigerator), but never at room temperature. 3) Matrigel was maintained at 4 °C or on ice during manipulation to prevent premature polymerization.

Reagents for organoid immunohistochemistry:

- Agarose (Pronadisa; cat. no. 8016)
- Cryomolds (Bio-Optica; cat. no. 07-MP7070)
- Single-edge razor blades (Trodis; cat. no. 26025)
- Tissue cassettes (VWR (Avantor); cat. no. 720-2186)
- Absolute ethanol (PanReac; cat. no. 141086.1211)
- 96% ethanol (Oppac; cat. no. 033TC0037)
- Xylene (Prolabo; cat. no. 28973.363)
- Microtome blades (Astelab; cat. no. MCUT-3508BOX)
- TOMO slides (Matsunami; cat. no. TOM-1190)
- Diamond pencil (RS-PRO; cat.no. 394-217)
- Trizma Base (Sigma; cat. no. T6066)
- EDTA (PanReac; cat. no. 131669)
- H_2_O_2_ (PanReac; cat. no. 141077.1211)
- NaCl (PanReac; cat. no. 141659.1211)
- 37% HCl (PanReac; cat. no. 141020.1611)
- Tween® 20 (Sigma; cat. no. P1379)
- Anti-EpCAM (Abcam; cat. no. ab223582)
- Anti-CK19 (Abcam; cat. no. ab52625)
- Envision anti-Rabbit (Dako; cat. no. K4003)
- DAB (+) (Dako; cat. no. K3468)
- Plastic staining jar (DeltaLab; cat. no. 19335)
- Glass staining jar (VWR; cat. no. 6319311)

Reagents for organoid gene silencing.

- Lipofectamine RNAiMAX (Invitrogen; cat. no. 13778075)
- Opti-MEM (Gibco; cat. no. 31985047)
- FBS (Gibco; cat. no A5256701)
- siRNAs (Sigma-Aldrich)

Reagents were used for protein extraction, Western blots, RNA extraction, qPCR, DNA extraction, and NGS analysis.

Standard reagents and protocols for cell analysis were used.

### 2.4 Equipment

- 24-well cell culture plates (Nunc™ Thermo Fisher Scientific; cat. no. 144530)
- Cryogenic vial, 1 mL (e.g., Thermo Fisher Scientific; cat. no. 377267)
- Pipettes P2, P20, P200, and P1000 (e.g., Thermo Fisher)
- Filtered tips (e.g., Thermo Fisher P20 (cat. no. 10731194), P200 (cat. no. 11782584), and P1000 (cat. no. 11749855))
- Gel-loading pipet tips (e.g., DeltaLab; cat. no. 200079R)
- Falcon 70-µm nylon cell strainer (Corning/Falcon; cat. no. 352350)
- Class-II biological safety cabinet (e.g., TELSTAR; cat. no. BIO-II-A)
- CO_2_ cell culture incubator (37 °C, humidified, 5% CO_2_) (e.g., FORMA SCIENTIFIC; cat. no. 3121)
- Refrigerated centrifuge capable of 200–1,800 × g at 4 °C (with adapters for 1.5-mL and 15/50-mL tubes)
- Water bath at 37 °C
- Tube rotator and/or rocker platform (required for steps performed under gentle agitation; e.g., rocking conducted at 32 °C during siRNA incubation)
- Inverted brightfield microscope for organoid monitoring (e.g., Zeiss; Cell Observer Z1) with a camera for image acquisition (e.g., Zeiss; Axiocam MRm).
- Rotary microtome (e.g., Microm; cat. no. HM 340E)
- Tissue flotation bath (e.g., MEDITE Medical GmbH; cat. no. TFB 35)
- Pascal pressure chamber (e.g., Dako; cat. no. S2800)
- Drying oven (e.g., INDE lab; cat. no. IDL-AL-36)
- A digital slide scanner (e.g., Aperio CS2 Digital Scanner, Leica Biosystems) is required to scan the immunohistochemistry preparations.

### 2.5 Reagent setup

#### 2.5.1 Reagents for the organoid culture mediums

All the reagents were prepared under sterile conditions and according to the manufacturers’ recommendations. Stock solutions were prepared at the concentrations indicated below, aliquoted to avoid repeated freeze–thaw cycles, and stored at the appropriate temperatures until use.

- Nicotinamide (1 M): 250 mg nicotinamide was dissolved in 2.05 mL sterile distilled water, mixed until fully dissolved, aliquoted, and stored at −80 °C.
- N-acetylcysteine (NAC, 500 mM): 250 mg of NAC was dissolved in 3.06 mL sterile distilled water, aliquoted, and stored at −20 °C for a maximum of 1 month.
- Y-27632 (ROCK inhibitor, 10 mM): 2 mg Y-27632 was dissolved in 624 µL sterile PBS, aliquoted, and stored at −80 °C.
- [Leu15]-Gastrin I human (100 µM): 0.1 mg peptide was dissolved in 480.7 µL PBS supplemented with BSA (PBS/BSA), aliquoted, and stored at −80 °C.
- Recombinant human HGF (25 μg/mL): 25 µg HGF was resuspended in 1 mL PBS/BSA, aliquoted, and stored at −80 °C.
- Recombinant human FGF10 (100 μg/mL): 25 µg FGF10 was dissolved in 250 µL PBS/BSA, aliquoted, and stored at −80 °C.
- Recombinant human EGF (50 μg/mL): 100 µg EGF was resuspended in 2 mL PBS/BSA, aliquoted, and stored at −80 °C.
- A83-10 (TGF-β inhibitor, 500 µM): 0.5 mg A83-10 was dissolved in 2.37 mL DMSO, aliquoted, and stored at −20 °C.
- Forskolin (8 mM): 10 mg forskolin was dissolved in 3.045 mL DMSO, aliquoted, and stored at −20 °C.

Basal culture medium (BCM):BCM consisted of Advanced DMEM/F-12 (500 mL) supplemented with 5 mL glutamine/penicillin/streptomycin (100× stock; final concentration 1×) and 5 mL HEPES (1 M stock; final concentration 10 mM). The medium was gently mixed and stored at 4 °C until use. BCM was used for the washing steps and as the base medium for preparation of complete culture medium (CCM).

CCM was prepared by supplementing BCM with Primocin (100 μg/mL), 3dGRO™ L-WNT conditioned medium (1×), N2 supplement (1×), B27 supplement without vitamin A (1×), nicotinamide (10 mM), NAC (1.25 mM), Y-27632 (10 µM), [Leu15]-Gastrin I (10 nM), recombinant human HGF (25 ng/mL), recombinant human FGF10 (100 ng/mL), recombinant human EGF (50 ng/mL), A83-01 (5 µM), and forskolin (10 µM). The final composition and the volumes required to prepare 10 mL CCM are detailed in Supplementary Table S1. CCM was gently mixed by inversion and used immediately or stored at 4 °C until use. CCM was prepared weekly. For using CCM, only the required volume was pre-warmed to 37 °C; the pre-warmed medium was not returned to storage at 4 °C, and any leftover medium was discarded.

10× ACK lysis buffer (1.5 M NH_4_Cl, 100 mM KHCO_3_, and 1 mM EDTA; pH 7.2–7.4; 500 mL): A 10× ACK lysis buffer stock solution was prepared by dissolving 43 g of NH_4_Cl, 5 g of KHCO_3_, and 0.186 g of disodium EDTA (EDTA-2Na) in approximately 400 mL of sterile distilled water with stirring until complete dissolution. The pH was adjusted to 7.2–7.4, and sterile distilled water was then added to a final volume of 500 mL. The solution was filter-sterilized through a 0.22-µm filter and stored at 4 °C.

1× ACK lysis buffer (0.15 M NH_4_Cl, 10 mM KHCO_3_, and 0.1 mM EDTA; pH 7.2–7.4): A 1× ACK working solution was prepared immediately before use by diluting the 10× stock in a 1:10 ratio in sterile distilled water.

#### 2.5.2 Reagents for organoid processing

2% agarose (w/v) in PBS: A 2% agarose solution was prepared by weighing 2 g of agarose and adding it to 100 mL of PBS. The suspension was heated in a microwave in several short intervals until boiling and complete dissolution. The solution was then allowed to cool to 55 °C–60 °C in a water bath before use.

Tris-EDTA buffer (10 mM Tris base and 1 mM EDTA; pH 9.0; 1 L). A Tris-EDTA (TE) buffer was prepared by dissolving 1.21 g of Trizma Base and 0.37 g of EDTA in 990 mL of deionized water with stirring until complete dissolution. The pH was checked and adjusted to 9.0 if necessary. Deionized water was then added to a final volume of 1 L. The buffer was stored at room temperature for up to 3 months.

5× Tris Buffered Saline (5× TBS; 0.25 M Tris base and 2.5 M NaCl; pH 7.36; stock solution): A 5× TBS stock solution was prepared by dissolving 30.26 g of Trizma Base and 145 g of NaCl in 850 mL of deionized water with stirring until fully dissolved. The pH was adjusted to 7.36 using 37% HCl (15 mL was added initially, followed by gradual addition until pH 7.36 was reached). Deionized water was added if needed to reach the desired final volume. The solution was stored at 4 °C for several months.

1× Tris Buffered Saline with Tween-20 (TBS-T; 0.05 M Tris base and 0.5 M NaCl; pH 7.36; 0.05% Tween-20; 1 L). A 1× TBS-T solution was prepared by diluting 200 mL of 5× TBS stock solution with 800 mL of deionized water. Tween-20 was added to a final concentration of 0.05% (500 µL per 1 L), and the solution was mixed thoroughly until it was homogeneous. The buffer was stored at 4 °C for several months.

RIPA buffer (150 mM NaCl, 50 mM Tris, 0.5% sodium deoxycholate, 0.1% SDS, and 1% Triton X-100, supplemented with phosphatase inhibitors and protease inhibitors). RIPA buffer was prepared by combining NaCl and Tris stock solutions with sodium deoxycholate, SDS, and Triton X-100 to obtain the indicated final concentrations. Immediately prior to use, the buffer was supplemented with a phosphatase inhibitor cocktail containing 1 mM sodium orthovanadate, 10 mM sodium fluoride, and 100 mM β-glycerophosphate, as well as protease inhibitors (Roche, Basel, Switzerland). The buffer was kept ice-cold and subjected to cell lysis for 20 min at 4 °C under constant rotation.

## 3 Methods

### 3.1 Establishment of patient-derived bile organoids

The following is a critical step. Once ERCP is completed, bile is stored at 4 °C and processed within 2 h of collection.

#### 3.1.1 Before beginning: duration: 30 min (plus ≥ 1 h of plate pre-warming)

Bile must be processed fresh. Before starting the protocol, the following steps must be followed:

- The culture plate where organoids will be seeded is pre-warmed for ≥ 1 h in a 37 °C incubator.
- The centrifuge is cooled to 4 °C.
- The Matrigel is thawed slowly on ice or at 4 °C. Slow thawing is critical to prevent bubble formation and premature gelation.
- CCM is prepared (prepare reasonable volumes and avoid storing complete medium for >1 week at 4 °C).

#### 3.1.2 Pre-culture processing of bile samples: duration ∼60 min–75 min

- Bile (4 mL–12 mL) collected at the endoscopic facility in sterile additive-free tubes is distributed into 1-mL aliquots and centrifuged for 10 min at 1,800 × g at 4 °C.

NOTE#2. The initial bile volume within the range analyzed did not appear to influence organoid establishment (see Results Section 4.1).

b. The supernatant is removed (bile can be kept for subsequent analyses), and the resulting pellet is resuspended from each 1-mL aliquot in 1 mL of cold BCM.

NOTE#3. A visible pellet is not always observed after centrifugation. Regardless, the subsequent steps should be performed as organoids can still develop even without a discernible pellet (see Results Section 4.1).

NOTE#4. If a relatively large yellow–brown loose pellet is observed, dark aggregates may be observed when seeding the drop (step n). It is important to continue with the protocol as, in our study, organoids were still successfully generated from some samples under these conditions.

c. All the BCM aliquots are filtered through a 70-µm nylon cell strainer placed over a conical 50-mL tube.
d. The conical 50-mL tube is centrifuged at 400 x g for 5 min at 4 °C.
e. The supernatant is discarded, and the pellet is washed, resuspended in 8 mL BCM, and centrifuged at 400 × g for 5 min at 4 °C, and the process is repeated two times (total of three washes).

#### 3.1.3 Erythrocyte lysis (if required): duration 10 min–15 min

The following is a critical Step. If a red pellet is observed after washing, the erythrocytes are lysed as follows:

f. A measure of 500 µL 1× ACK lysis buffer is added to the pellet, and the tube is flicked to dislodge the pellet. It is then incubated for 2 min at 37 °C.
g. The reaction is stopped by adding 2.5 mL of cold BCM (5× the ACK volume).
h. After centrifugation for 5 min at 400 × g at 4 °C, the supernatant is discarded.

* If the pellet remains clearly red, the ACK lysis step (f–h) is repeated.

NOTE#5. Here, ACK lysis did not appear to reduce organoid establishment (see Results Section 4.1). Nevertheless, longer or repeated lysis may compromise sample integrity; therefore, in our workflow, we avoided performing more than two consecutive ACK lysis cycles on the same sample and proceeded even if a slight red tinge persisted.

#### 3.1.4 Matrigel embedding and culture initiation: duration ∼45 min–60 min

i. The pre-warmed untreated 24-well plate is retrieved from the incubator and placed in the biosafety cabinet.
j. The tube from step h is taken, and it is ensured that the supernatant is completely removed. Cold Matrigel is added according to the number of domes to be prepared (35 µL per dome), and the pellet is rapidly but gently resuspended using pre-chilled, cut tips.

The following is a critical step. Matrigel is stored at 4 °C or on ice during handling; single-use aliquots are used to avoid repeated freeze–thaw cycles.

NOTE#6. Typically, one dome is prepared if no visible pellet is observed, and two domes are prepared if a visible pellet is obtained.

k. The 35-µL domes of the Matrigel–cell suspension are dispensed onto the center of a well of the pre-warmed plate.

NOTE#7. Volumes >35 µL may reduce dome integrity and roundness.

l. The plate is immediately inverted and incubated for 35 min–40 min at 37 °C to allow polymerization.

The following is a critical step: Inverting the plate prevents cells from settling at the bottom of the plate and helps ensure uniform embedding within the Matrigel.

m. The plate is carefully returned to the upright position, and 600 µL of pre-warmed CCM is added per well.
n. The organoids are maintained at 37 °C in a humidified atmosphere with 5% CO_2_.

### 3.2 Organoid maintenance and expansion

- The organoids are inspected under brightfield microscopy at least twice per week, and the morphology is documented (e.g., cystic vs. filled/compact organoids and presence of darkening/debris).
- CCM is replaced twice per week using pre-warmed medium.

**FIGURE 1:**
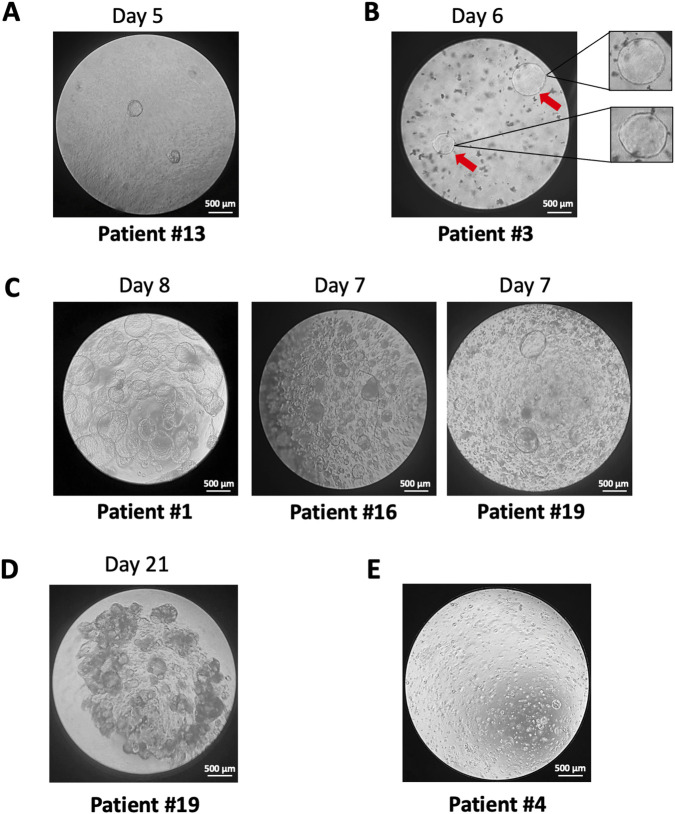
Representative brightfield microscopy of bile-derived organoid cultures. Brightfield microscopy at ×4 magnification illustrates the developmental stages and culture quality of organoids derived from different patients. **(A)** Initial organoid growth observed at day 5 post-collection (patient #13). **(B)** Organoid formation at day 6 (patient #3) from a sample presenting a large yellow–brown loose pellet, with organoids emerging amid dark aggregates. **(C)** Representative images from three distinct patients showing a high density of large, well-formed organoids at optimal confluence for passaging. **(D)** Morphological darkening of organoid structures, typically indicating reduced viability or the onset of cell death. **(E)** Established organoids 3 days post-passaging displaying exponential growth phase and readiness for cryopreservation.

NOTE#8. Organoid outgrowth duration and establishment efficiency may vary across samples. In our hands, the initial organoid outgrowth is typically observed within 2–5 days (Figure 1A).

NOTE#9. Figure 1B shows representative organoids at day 6 post-seeding from cultures initiated under the conditions described in NOTE#3, presenting debris or dark aggregates.

NOTE#10. After successful establishment, users may consider short “maturation windows” by reducing WNT3A/R-spondin stimulation and selected growth factors to favor a more differentiated cholangiocyte phenotype, while maintaining standard CCM for routine expansion and biobanking (Huch et al., 2015; Sampaziotis et al., 2017; Roos et al., 2021a).

### 3.3 Organoid passaging: duration ∼1.5 h

NOTE#11. Organoids are passaged when the Matrigel dome is densely populated with large, well-formed organoids (see Figure 1C for representative brightfield images). A typical split ratio is 1:3.

NOTE#12. Progressive darkening of organoids often indicates reduced viability and/or onset of cell death. Here, this typically reflects overgrowth and indicates that the organoids should have been passaged earlier (see Figure 1D for representative brightfield images).

CCM is removed from the wells that are going to be split. If multiple domes from the same patient/condition are split on the same day, we recommend pooling them and then generating the corresponding number of domes.

- CCM is removed from wells to be passaged.
- A measure of 500 µL of ice-cold BCM is added to the well, and the Matrigel dome is gently disrupted using circular pipetting movements. This step is repeated twice to maximize recovery. The suspension is then transferred into a 15-mL conical tube containing 10 mL ice-cold BCM.

NOTE#13. If multiple domes from the same patient/condition are passaged on the same day, all the domes are pooled into the same 15-mL conical tube.

c. Centrifuge at 600 × g for 5 min at 4 °C. The supernatant is carefully removed without disturbing the pellet.
d. Enzymatic digestion (TrypLE): 500 μL to 1 mL TrypLE is added, depending on the pellet size (adapted from (Driehuis et al., 2020)).

NOTE#14. As a guideline, 1 mL TrypLE is typically used to digest material from >3 Matrigel domes containing multiple organoids.

e. The tube is gently tapped to detach organoids from the bottom. It is then incubated for 3 min at 37 °C in a water bath.
f. The organoids are mechanically disrupted by pipetting up and down 10 times.
g. Digestion is stopped by adding 5× the TrypLE volume of BCM.
h. After centrifugation at 300 × g for 5 min at 4 °C, the supernatant is discarded.
i. The pellet is resuspended in the corresponding volume of Matrigel (according to the number of domes to be prepared according to the original number of domes harvested; 35 µL per dome), and the domes are plated onto a pre-warmed 24-well plate (step j in 3.1.4.). The plate is immediately inverted and incubated for 35 min–40 min at 37 °C to allow polymerization.
j. The plate is returned to the upright position, and 600 µL of pre-warmed CCM is added per well.

### 3.4 Cryopreservation and thawing of organoids

The following protocols are adapted from the methodology described by Driehuis et al. (2020).

#### 3.4.1 Cryopreservation: duration ∼30 min–45 min

The following is a critical step: organoids should be frozen 3–5 days post-passaging to ensure they are in exponential-phase growth; this improves post-thaw survival and their ability to resume growth after thawing (see Figure 1E for representative brightfield images of organoids at the time of freezing).

NOTE#15. We typically freeze one Matrigel dome per cryovial. If multiple domes are harvested on the same day, they may be pooled and then aliquoted into as many cryovials as the number of domes originally collected.

- The Matrigel domes are mechanically disrupted by adding 500 µL of ice-cold BCM per well. Pipetting with gentle circular motions is carried out until the domes are fully detached and the organoids are evenly suspended.
- The disrupted domes are transferred to a single pre-chilled 15-mL conical tube, pooling the material from different domes.
- Centrifugation is carried out at 300 x g for 5 min at 4 °C.
- The supernatant is carefully aspirated without disturbing the organoid pellet.
- A measure of 500 µL of Recovery™ Cell Culture Freezing Medium is added per Matrigel dome originally harvested.
- The pellet is gently resuspended in the freezing medium and dispensed into cryovials.
- The cryovials are immediately placed at −80 °C. After 3–4 days, the vials are transferred to liquid nitrogen for long-term storage.

#### 3.4.2 Thawing: duration ∼1 h

The following is a critical step: rapid thawing is essential to maintain organoid viability. The transition from liquid nitrogen to 37 °C must be as fast as possible.

NOTE#16. Cryopreserved bile-derived organoid cultures could be successfully recovered after up to 175 days of cryopreservation. Longer-term storage performance requires further evaluation (see Results Section 4.2).

- The cryovial is retrieved from storage and immediately placed in a 37 °C water bath. The vial is held until only a small ice crystal remains.
- Under sterile conditions, gentle “up and down” pipetting is performed to resuspend the organoids.
- The suspension is transferred to a 15-mL conical tube, and an additional 4 mL of BCM is added.
- Centrifugation is carried out at 300 x g for 5 min at 4 °C.
- The supernatant is aspirated completely.
- The organoid pellet is resuspended in the appropriate volume of Matrigel and seeded as droplets (35-µL domes, step j in 3.1.4.) onto a 37 °C pre-warmed culture plate.
- The plate is immediately inverted and incubated for 35 min–40 min at 37 °C to allow polymerization.
- The plate is carefully returned to the upright position, and 600 µL of pre-warmed CCM is added per well.
- Organoids are maintained at 37 °C in a humidified atmosphere with 5% CO_2_.

### 3.5 Gene expression knockdown in bile-derived organoids

#### 3.5.1 Gene silencing in dissociated organoids: duration ∼7 h

SCOPE: Organoids are dissociated to a near single-cell suspension, and gene expression knockdown is performed prior to re-embedding, enabling the assessment of the effects of gene silencing on organoid initiation and formation.

NOTE#17. In this study, gene silencing is performed in 24-well plates (final volume 1 mL/well) under gentle rocking at 32 °C for 5 h.

- Pre-formed organoids are collected and dissociated using the TrypLE-based digestion procedure described in Section 3.3 (organoid passaging), while adapting volumes as needed to obtain a near single-cell suspension that is suitable for counting.

The following is a critical step. Efficient Matrigel removal and generation of a homogeneous suspension in a suitable volume for accurate cell counting are essential for reproducible seeding and gene silencing.

b. The cells are resuspended and counted using a Neubauer hemocytometer. A total of 50,000 cells per condition are placed in 800 µL Opti-MEM + 10% FBS in 24-well plates.

NOTE#18. To minimize variability, a single master cell suspension is prepared in Opti-MEM + 10% FBS containing the total number of cells required for all wells, along with a small excess to account for pipetting losses. For example, to seed six wells at 50,000 cells/well in 500 µL/well, a master mix of 3.2 mL containing 320,000 cells (equivalent to 100,000 cells/mL) is prepared, and 500 µL is dispensed per well. The suspension is mixed gently but thoroughly before dispensing each aliquot to ensure homogeneous cell distribution.

c. siRNA transfection mixes are prepared using lipofectamine RNAiMAX according to the manufacturer’s instructions. For each siRNA condition, a single master siRNA–RNAiMAX mix is prepared in 200 µL Opti-MEM per well containing siRNA at 50 nM (plus ∼5%–10% excess for pipetting losses). A measure of 200 µL of the corresponding siRNA–RNAiMAX mix is added to each well that already contains 800 µL of the cell suspension. This results in a final volume of 1 mL per well and a final siRNA concentration of 10 nM.
d. Plates are incubated at 32 °C for 5 h under gentle agitation on a rocking platform placed inside the incubator.

The following is a critical step. During incubation, cells should remain in suspension to maximize siRNA uptake and prevent cell attachment. Low-attachment plates are used, along with a relatively large working volume (1 mL/well) and gentle rocking throughout the incubation.

e. After 5 h, the cells are collected and centrifuged at 200 × g for 5 min at 4 °C. The supernatant is carefully removed without disturbing the cell pellet.
f. The cell pellet is reembedded in Matrigel, and culture is restarted as described in steps h–i in Section 3.3 (Organoid passaging).

#### 3.5.2 Gene silencing in fully formed organoids: duration ∼7 h

SCOPE. Pre-formed organoids are silenced while embedded/maintained as 3D structures, enabling assessment of the effects of gene silencing in established organoids (e.g., viability, growth, morphology, and downstream molecular readouts).

NOTE#19. In this study, gene silencing is performed in 1.5-mL conical tubes (final volume 1 mL/tube) under gentle rocking at 32 °C for 5 h.

- Without removing the culture medium from the well, the Matrigel dome containing organoids is carefully retrieved from the culture well without disrupting its structure. It is transferred into a conical-bottom 1.5-mL tube containing 700 µL cold PBS. A small sterile spatula or spoon-shaped lifter can be used to handle the dome safely.

NOTE#20. Optionally, two domes from the same condition can be combined into one tube (do not exceed two). However, processing domes separately is recommended whenever possible as it facilitates more uniform Matrigel disruption and handling and may improve the overall silencing efficiency.

b. Centrifugation is carried out for 1 min at 200 × g at 4 °C to settle the Matrigel dome without deforming it. PBS is carefully removed.

The following is a critical step. To avoid aspirating organoids, PBS is removed using a 200-µL gel-loading tip.

c. The organoids are washed by adding 700 µL cold PBS and gently pipetting up and down four times using a cut P1000 tip to remove residual Matrigel while maintaining organoid integrity.
d. Centrifugation is carried out again (1 min at 200 × g, 4 °C), and all of the PBS is carefully removed.
e. A measure of 800 µL Opti-MEM + 10% FBS is added in two steps; first, 2 × 200 μL is added, the organoids are gently detached from the residual Matrigel with 2–3 gentle up-and-down pipetting using a cut tip, and then the remaining 400 µL is added.

The following is a critical step. Avoid vigorous pipetting should be avoided to preserve the organoid structure.

f. siRNA transfection mixes are prepared using Lipofectamine RNAiMAX according to the manufacturer’s instructions. For each condition, an siRNA–RNAiMAX mix is prepared in 200 µL Opti-MEM containing siRNA at 100 nM (plus ∼5%–10% excess if preparing multiple tubes). A measure of 200 µL of this mix is added to each tube already containing 800 µL Opti-MEM + 10% FBS (step e). This yields a final volume of 1 mL per tube and a final siRNA concentration of 20 nM.
g. The tubes are incubated at 32 °C for 5 h under continuous gentle agitation on a rocking platform placed inside the incubator. The tubes are secured to prevent tipping (e.g., 1.5-mL tubes are placed inside a 50-mL conical tube cushioned with paper towels).

The following is a critical step. Continuous gentle agitation improves the exposure of organoids to the silencing mix and helps maintain uniform conditions within the tube.

h. After 5 h, the suspension is centrifuged for 1 min at 200 × g at 4 °C. The siRNA-containing medium is carefully discarded without aspirating the organoids.
i. The organoids are reembedded in Matrigel, and the culture is restarted, as described in steps h–i in Section 3.3 (Organoid passaging).

### 3.6 Immunohistochemistry of organoids: duration: 4 days

- Without removing the culture medium from the well, the Matrigel dome containing the organoids is carefully retrieved from the culture well without disrupting its structure. It is transferred into a conical-bottom 1.5-mL tube containing 200 µL of cold PBS. A small sterile spatula or spoon-shaped lifter can be used to handle the dome safely.

The following is a critical step. PBS is added to the 1.5-mL tube before transferring the organoids. The buffer facilitates organoid release from the spatula and prevents organoids from sticking to the plastic.

NOTE#21. Try to collect the entire droplet without disrupting it. If most of it can be recovered on the first attempt, repeating the process should be avoided, as attempting to collect it again may disrupt the organoids.

b. The volume is adjusted to 1 mL with PBS, and the tube is gently inverted 3–5 times to wash the sample.
c. After centrifugation for 1 min at 200 × g at 4 °C, the supernatant is carefully removed.

NOTE#22. To minimize the risk of aspirating organoids, the supernatant is removed using a 200-µL gel-loading tip.

d. A measure of 1.2 mL of 4% paraformaldehyde (PFA) is added, ensuring complete coverage of the sample. It is fixed for 1 h at room temperature: it is incubated for the first 30 min under gentle rotation, then rotation is stopped, and the tube is left upright for an additional 30 min at room temperature to allow the organoids to settle at the bottom.

NOTE#23. To enable gentle and uniform rotation, the 1.5-mL tube is placed inside a 50-mL conical tube and stabilized (e.g., between two layers of paper). The 50-mL tube is mounted on a roller/rotator designed for conical tubes.

e. The PFA solution is carefully removed.
f. The organoids are washed (×3). A measure of 700 µL PBS is added, the tubes are gently inverted to resuspend, centrifugation is carried out for 2 min at 200 × g at 4 °C, and the supernatant is removed as in step (c). The process is repeated for a total of three washes.

PAUSE POINT. After the three PBS washes (step f), fixed organoids can be kept in PBS at 4 °C for up to 72 h before proceeding to agarose pre-embedding and paraffin processing. Although we recommend completing the workflow without interruption when possible, this pause point enables flexible scheduling.

g. A 2% agarose solution is prepared by heating it to boiling temperature in a microwave oven (or by melting a prepared stock solution).
h. After centrifugation for 5 min at 200 × g, the supernatant is removed. The 1.5-mL tube is placed in a water bath at 55 °C–60 °C.

NOTE#24. Keeping the tube in the hot water bath prevents the agarose from solidifying prematurely.

i. A measure of 200 µL of 2% agarose is gently added using a cut P1000 tip, and the organoids are mixed carefully.
j. The organoids/agarose mixture is gently but rapidly transferred into the cryomold.

NOTE#25. Placing the cryomolds on a dark surface helps visualize the transfer of organoids from the tube to the cryomold.

k. The cryomolds are kept at 4 °C until the agarose is completely solidified (∼20 min).
l. The solidified agarose containing the embedded organoids is carefully removed using a new razor blade and placed in a pre-labeled tissue cassette.
m. The tissue cassette is transferred to 70% ethanol until the subsequent paraffin embedding process.
n. The paraffin embedding workflow is carried out following a standard protocol for formaldehyde-fixed, paraffin-embedded (FFPE) samples.
o. The paraffin block is carefully trimmed and cut into 3-µm sections using a rotary microtome. The ribbon of sections is floated on a 42 °C water bath and collected onto adhesive microscope slides (TOMO slides were used). The slides were kept in a vertical position until completely dry (overnight). The dried sections can then be used for hematoxylin–eosin (H&E) staining and immunohistochemistry.

NOTE#26. Organoids are often difficult to visualize by the naked eye in the sections; therefore, their presence should be confirmed using a light microscope. It is advisable to gently mark the position of the sections on the back of the slide with a diamond pencil to facilitate subsequent localization.

p. Slides are baked in a 60 °C drying oven for 30 min. Running tap water is used to deparaffinize and rehydrate the sections.
q. Antigen retrieval is performed using TE buffer (pH 9) in a Pascal Pressure Chamber at 95 °C for 30 min. The slides were allowed to cool for 25 min at room temperature.
r. Endogenous peroxidase was blocked by incubating the slides in 3% H_2_O_2_ in distilled water for 12 min at room temperature and briefly rinsing in running tap water.
s. The slides were washed in TBS-T for 5 min.
t. The slides surrounding the tissue sections were gently dried, and the primary antibodies anti-EpCAM (Abcam, ab223582; 1:1,000) or anti-CK19 (Abcam, ab52625; 1:200) were added.
u. The slides were incubated overnight at 4 °C in a humidified chamber.
v. The slides are washed in TBS-T for 5 min.
w. The slides are dried around sections and incubated with peroxidase-labeled polymer-conjugated goat anti-rabbit (EnVision+). They are incubated for 30 min at room temperature.
x. After washing in TBS-T for 5 min, DAB + solution is added for 10 min. They are rinsed with running tap water for 5 min.
y. Finally, the sections are lightly counterstained with Harris hematoxylin, followed by dehydration and mounting.

### 3.7 Protein extraction and western blot analysis: duration ∼36 h

- Organoids are collected from Matrigel, and a cell suspension is obtained as described in steps a–g in Section 3.3 (organoid passaging), adapting volumes as needed.
- Cells are lysed in ice-cold RIPA buffer (see Reagent setup) supplemented with protease and phosphatase inhibitors for 20 min at 4 °C under constant rotation.

The following is a critical step. Keep samples and buffers are kept cold to preserve protein integrity and phosphorylation states.

c. Lysates are sonicated and centrifuged at 11,000 g for 20 min at 4 °C to remove the insoluble material. The clarified supernatant is transferred to a new tube.
d. The protein concentration is measured using the BCA assay (Pierce) according to the manufacturer’s instructions.
e. Western blot analysis is performed as previously described (Jiménez et al., 2019).

### 3.8 RNA isolation and molecular analyses

#### 3.8.1 RNA isolation: duration ∼1 h

- Organoids are collected, and a cell pellet is obtained as described in Section 3.3 (Organoid passaging, steps a–g), stopping after the final centrifugation step (prior to Matrigel re-embedding).
- RNA is extracted using the automated Maxwell RSC Instrument with the Maxwell RSC simply RNA Tissue kit (Promega, Madison, WI, United States of America) following the manufacturer’s instructions.
- The RNA concentration is measured using a NanoDrop spectrophotometer, as appropriate for downstream applications.

#### 3.8.2 Real-time PCR: duration ∼3 h

d. Real-time PCRs were performed with the iQ SYBR Green Supermix (Bio-Rad, Hercules, CA, United States of America) in a CFX96 Real-Time System (Bio-Rad), as previously described (Urtasun et al., 2016).

### 3.9 Total DNA isolation and molecular analyses

#### 3.9.1 Total DNA isolation: duration ∼1 h–2 h

- Organoids are collected, and a cell pellet is obtained as described in Section 3.3 (Organoid passaging, steps a–g), stopping after the final centrifugation step (prior to Matrigel re-embedding).
- Total DNA was extracted using the automated Maxwell RSC Instrument with the Cultured Cells DNA kit (Promega, Madison, WI, United States of America) following the manufacturer’s instructions.
- DNA concentration was measured using a NanoDrop spectrophotometer and/or Qubit, as appropriate, for downstream applications.

#### 3.9.2 Next-generation sequencing DNA analyses

Targeted sequencing was performed using 50 ng of DNA with the Oncomine™ Pan-Cancer Cell-Free Assay (Thermo Fisher Scientific, Waltham, MA, United States of America) according to the manufacturer’s instructions and as previously described (Arechederra et al., 2022; 2025). This targeted panel investigates the hotspot mutations and short insertions/deletions across 52 cancer-related genes.

#### 3.9.3 ULP-WGS for copy number alterations (CNAs)

Ultra-low-pass whole-genome sequencing (ULP-WGS) was performed using 5 ng of DNA, as previously described (Sogbe et al., 2023) and according to the manufacturer’s instructions, where applicable. Copy-number profiling and tumor fraction (TF) estimation were conducted using the ichorCNA framework, following the recommended workflow for cfDNA to infer large-scale CNAs and aneuploidy patterns (Adalsteinsson et al., 2017).

## 4 Results

### 4.1 Cohort description and organoid establishment rate

Bile samples were collected from 21 individuals undergoing ERCP for biliary strictures (Table 1). The cohort comprised five cases with benign biliary stenosis due to choledocholithiasis and 16 cases with a malignant cause of stricture, including 13 CCAs, 1 gallbladder adenocarcinoma, and 2 ampullary tumors.

Successful establishment was defined as the generation of organoids that could be expanded and passaged at least once. Using the workflow described in Section 3.1, bile-derived organoids were successfully established in 17/21 samples (81%) (Table 2). We first examined whether the establishment success was associated with sample processing variables. The initial bile volume was variable across the samples and did not appear to predict success. In established cases (n = 17), the mean initial volume was 8 mL (range, 4 mL–11 mL), while among non-established cases, three samples had an initial volume of 8 mL and one had an initial volume of 14 mL. Likewise, the absence of a visible pellet after centrifugation was not incompatible with organoid establishment as organoids were successfully generated in six of nine (67%) samples in which no visible pellet was observed. ACK lysis was required in five of the 21 bile samples, and all five gave rise to successfully established organoids, indicating that the need for ACK lysis was not associated with reduced establishment success in our cohort. Finally, regarding the relationship between the establishment rate and patient diagnosis (Table 2), given the small number of cases in some groups, the observed differences should be interpreted with great caution. Success rates were 85% (11/13) for CCA, 100% (2/2) for ampullary tumor, 100% (1/1) for GBC, and 60% (3/5) for choledocholithiasis.

**TABLE 2:** Establishment success of bile-derived organoids according to the diagnosis.

Diagnosis	Established	Non-established	Total	Success rate (%)
Choledocholithiasis	3	2	5	60
Cholangiocarcinoma	11	2	13	85
Gallbladder cancer	1	0	1	100
Ampullary tumor	2	0	2	100

The study cohort (n = 21) consisted of five cases of benign stenosis (choledocholithiasis) and 16 malignant cases, including 13 CCA, one GBC, and two ampullary tumors. The percentage of establishment success per diagnostic group is defined as cultures successfully reaching at least one passage.

### 4.2 Morphology, outgrowth kinetics, and routine expansion

Of the successfully established cultures, organoids from seven patients (two choledocholithiasis, four CCA, and one GBC) were expanded and characterized in detail in this study. Brightfield microscopy revealed the appearance of epithelial structures compatible with biliary organoid outgrowth within ∼2–3 weeks after seeding. The time to first detectable organoid outgrowth varied across samples [range (4–19) days; median (11.71) days], which is consistent with interindividual variability in bile cellular content and sample quality (examples in Table 3).

**TABLE 3:** Time to establishment of bile-derived organoids.

Patient ID	Diagnosis	Time to establishment (days)
#1	Choledocholithiasis	4
#18	Choledocholithiasis	19
#13	CCA	16
#14	CCA	12
#15	CCA	10
#19	CCA	10
#16	GBC	11

Time to establishment (days) for the subset of seven patients (two choledocholithiasis, four CCA, and one GBC) characterized in detail in this study.

**FIGURE 2:**
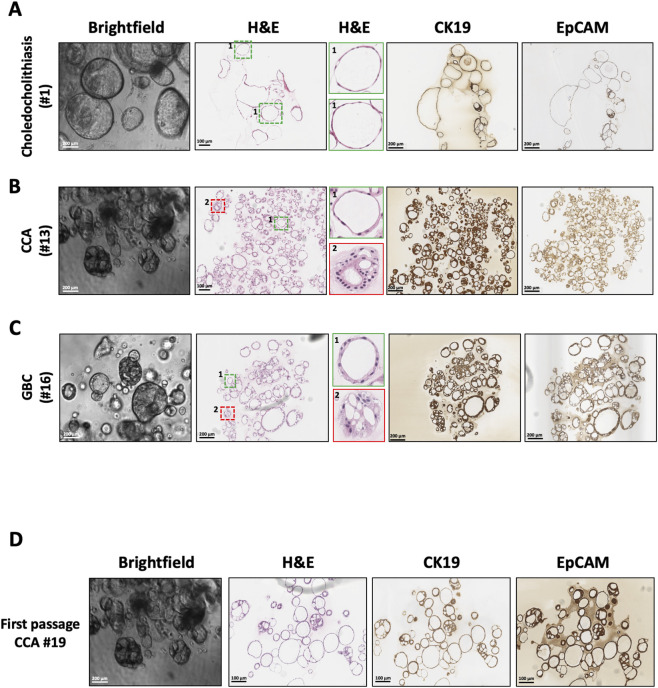
Morphology and histological characterization of bile-derived biliary organoids and culture drift during expansion. **(A)** Representative brightfield images and corresponding H&E, CK19, and EpCAM immunohistochemistry from a choledocholithiasis-derived culture (patient #1) showing typical cystic cholangiocyte-like organoids. **(B,C)** Representative brightfield images and corresponding H&E, CK19, and EpCAM immunohistochemistry from one CCA-derived culture (patient #13) **(B)** and one GBC-derived culture (patient #16) **(C)**, illustrating the coexistence of normal cholangiocyte-like and more tumor-like organoid populations within the same culture. Insets show magnified views of representative organoids: normal cholangiocyte-like organoids (1) are outlined in green, and tumor-like organoids (2) are outlined in red; note the larger size and increased structural complexity of the tumor-like structures. **(D)** Example of phenotypic drift during routine passaging of a CCA-derived culture (patient #19), illustrating a shift toward a predominantly cystic, cholangiocyte-like morphology over expansion (top: first passage; bottom: third passage).

In the choledocholithiasis-derived cultures, organoids displayed a typical cystic morphology, forming a single layer of epithelial cells positive for CK19 and EpCAM surrounding a central lumen (Figure 2A). Across samples from individuals with malignant strictures, we observed marked intra- and inter-sample heterogeneity in organoid morphology. In most cases, cultures initially contained mixed organoid populations of normal cholangiocyte-like and tumoral-like organoids (Figures 2B,C). Representative brightfield images and H&E, CDK19, and EPCAM immunohistochemistry of the organoids obtained from a CCA patient (Figure 2B) and a GBC patient (Figure 2C) are shown. In the H&E staining images, organoids with normal (1) and tumoral (2) morphology are shown at higher magnification.

However, under routine expansion and passaging, the cultures often drifted over time toward an almost exclusive cystic phenotype, which is consistent with the preferential outgrowth of non-malignant cholangiocyte-derived organoids (Figure 2D). Therefore, physical separation by manual handpicking, as previously described (Broutier et al., 2017), would be required to isolate and maintain the tumor-derived organoid population.

After establishment and expansion, cryopreserved organoid cultures were thawed to assess post-thaw recovery rates. A total of six thawing attempts were performed on cryopreserved cultures derived from four patients (Supplementary Table S2). In five of the six thawed vials, organoids resumed growth after thawing and re-embedding in Matrigel. The only unsuccessful thawing attempt was observed for a vial that had remained cryopreserved for 700 days, which is the longest period tested. However, as it is an isolated case, it cannot be assumed that the failure to recover cultured organoids is due to the effects of prolonged cryopreservation. Nevertheless, in our study, bile-derived organoid cultures were successfully recovered after up to 175 days of cryopreservation.

### 4.3 Pilot targeted NGS and ULP-WGS in selected paired bile–organoid samples

To evaluate whether bile-derived organoids capture the genomic features detected in the matched bile samples, we performed targeted NGS using the Oncomine™ Pan-Cancer Cell-Free Assay in selected paired bile–organoid samples (Table 4). As expected, in a choledocholithiasis control (patient #1), no mutations were detected in either bile cfDNA or the matched organoid culture. However, the same *TP53* polymorphic variant (p.R213 =) was detected in both samples with comparable MAF values (52.21% and 47.32%, respectively), which is consistent with a germline polymorphism of the patient. In a representative CCA case (patient #13), two mutations at low MAF in *EGFR* (R451C; 0.08%) and *KRAS* (G12D; 0.02%) were detected in bile cfDNA. In the matched organoid culture, the *KRAS* mutation (G12D; 0.12%) was also detected, whereas the *EGFR* mutation (R451C) was not detected. In addition, this case also harbored the *TP53* p.R213 = polymorphic variant in both bile and organoid DNA. In a representative GBC case (patient #16), bile cfDNA sequencing identified a low-allele-frequency *ERBB3* mutation (D297Y; 0.015%). The matched organoid culture harbored the same variant at a higher MAF (D297Y; 1.11%). Overall, these results indicate that targeted sequencing is feasible in organoid-derived DNA and can recover bile-detected alterations.

**TABLE 4:** Targeted NGS in selected paired bile–organoid samples.

​	Sample	NGS mutational analysis (PanCancer)	
Patient ID	Diagnosis	Bile	Bile-derived organoids
#1	Choledocholithiasis	*TP53 polymorphism* (R213; 52,2141%)	*TP53 polymorphism* (R213; 47,3203%)
#13	CCA	*KRAS* (G12D; 0.02%); *EGFR* (R451C; 0.0788%); *TP53 polymorphism* (R213; 45,5474%)	*KRAS* (G12D; 0.1272%); *TP53 polymorphism* (R213; 49.8686%)
#16	GBC	*ERBB3* (D297Y; 0.015%)	*ERBB3* (D297Y; 1.1178%)

List of mutations identified in the paired bile cfDNA and bile-derived organoid gDNA using the Oncomine™ Pan-Cancer Cell-Free Assay. Gene, amino acid change, and the percentage of mutant-allele frequency are indicated. Abbreviations: cfDNA: cell-free DNA; gDNA: genomic DNA.

In addition, we assessed the genome-wide copy number alterations by ULP-WGS in selected paired samples (Table 5). In the choledocholithiasis control (patient #1), TF estimates were 0 in both bile and organoid-derived DNA, as expected for a non-malignant condition. In contrast, in a CCA case (patient #14), ULP-WGS revealed a detectable TF of 39% in bile and a lower TF of 8.4% in the corresponding organoid culture, supporting the feasibility of CNA profiling from both bile and bile-derived organoids and indicating that organoid-derived DNA can retain tumor-associated signals that are detected in bile.

**TABLE 5:** ULP-WGS in selected paired bile–organoid samples.

​	Sample	ULP-WGS	
Patient ID	Diagnosis	Bile	Bile-derived organoids
#1	Choledocholithiasis	0	0
#14	CCA	39%	8.40%

Percentage of tumor fraction detected by ULP-WGS analyses by ichorCNA in the paired bile cfDNA and bile-derived organoid gDNA. Abbreviations: cfDNA: cell-free DNA; gDNA: genomic DNA.

### 4.4 siRNA gene expression knockdown in dissociated and formed organoids

We next evaluated the feasibility of siRNA-mediated gene silencing using two complementary workflows: silencing in dissociated organoids prior to re-embedding (Section 3.5.1) and silencing in fully formed organoids (Section 3.5.2).

**FIGURE 3:**
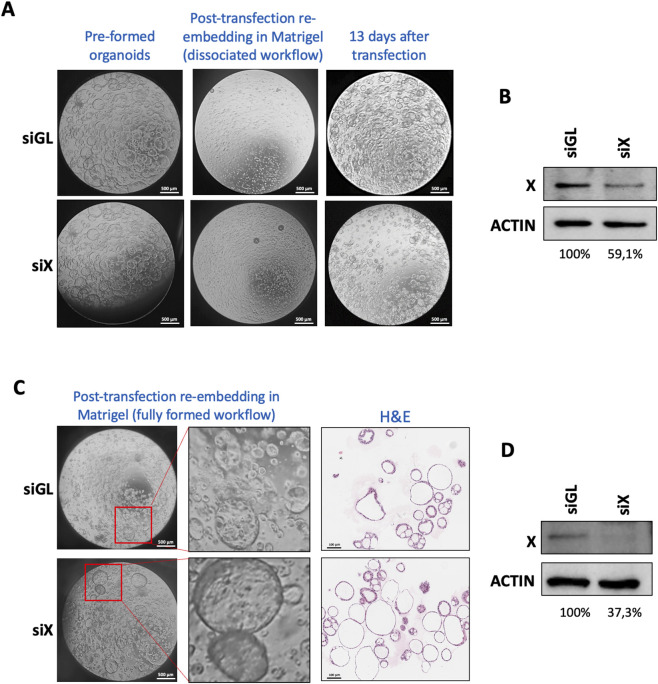
Evaluation of siRNA-mediated silencing in dissociated and fully formed bile-derived organoids. Representative brightfield microscopy and protein expression analysis demonstrate the knockdown efficiency across two transfection workflows. **(A)** Brightfield images of the dissociated organoid silencing workflow, showing pre-formed organoids, post-transfection re-embedding in Matrigel, and organoid reconstitution 13 days after transfection with either siRNA targeting the gene of interest (siX) or a non-targeting control (siGL). **(B)** Western blot analysis of protein X levels 96 h post-transfection in the dissociated workflow, with actin serving as a loading control. **(C)** Brightfield and immunohistochemical analysis of the fully formed organoid silencing workflow 72 h post-transfection with siRNA targeting the gene of interest (siX) or a non-targeting control (siGL). **(D)** Western blot analysis of protein X levels 96 h post-transfection in the fully formed workflow, with actin serving as a loading control.

In the dissociated workflow, we followed the protocol described in Aloia et al. (2019). Pre-formed organoids (Figure 3A) were dissociated to a single-cell-enriched suspension, transfected with siRNAs, and re-embedded in Matrigel (Figure 3A). As a proof of concept, we targeted X-mRNA using siX, with siGL as a negative control. After re-embedding, parallel wells were processed at defined time points for knockdown validation (96 h), while replicate wells were maintained for long-term monitoring of organoid re-formation and outgrowth (13 days). At 96 h post-transfection, the X protein levels were assessed by Western blot and were found to be reduced compared with that in control conditions (Figure 3B). In addition, qPCR analysis confirmed X-mRNA silencing in siX in this condition (Supplementary Figure S1A). Although at a different density, newly formed organoids were observed by day 13 in both the siGL control and the siX condition, indicating preservation of organoid-forming capacity following this procedure (Figure 3A).

In the fully formed organoid workflow, pre-formed organoids (Figure 3C) were transfected with siX, while using siGL as a control. At 72 h after transfection, histological assessment (H&E) supported the maintenance of organoid structural integrity following the silencing procedure (Figure 3C). Knockdown efficiency was evaluated at 96 h by Western blot (Figure 3D), showing reduced X protein levels compared with control siRNA conditions (siGL). qPCR analysis also confirmed X-mRNA silencing in fully formed organoids (Supplementary Figure S1B).

### 4.5 Pitfalls, limitations, and management

This protocol enables the generation and downstream manipulation of bile-derived organoids from clinically accessible samples; however, it is important to explicitly acknowledge several current limitations, together with the practical pitfalls and management considerations across the workflow.

First, bile samples are inherently heterogeneous, and establishment efficiency may vary depending on factors such as cellular content, sample quality, and diagnosis. Second, in cultures derived from malignant strictures, normal-like and tumor-like organoids may initially coexist, and routine expansion may favor the outgrowth of non-malignant cholangiocyte-like organoids, which can limit tumor-specific applications unless additional enrichment strategies are applied. Third, the amount of material obtained from the organoids is often limited, which may constrain downstream analyses and restrict the extent of validation and studies that can be performed in each case (IHQ, Western blot, RT-PCR, DNA sequencing, etc). Finally, some downstream applications presented here, including molecular profiling and gene-silencing workflows, should be interpreted as proof-of-concept examples rather than comprehensive benchmarking across large cohorts. The common pitfalls, protocol limitations, and recommended management measures are summarized in Supplementary Table S3.

## 5 Discussion

Robust human experimental systems are essential to move from descriptive studies toward actionable biology and translational testing. In the biliary tract, model availability has lagged behind other tissues, largely because access to healthy cholangiocytes is limited and tissue sampling is invasive, scarce, and often biased toward late-stage or surgically resected material. Tissue-derived cholangiocyte organoids were enabled by long-term expansion of adult human liver EpCAM + ductal cells in organoid culture conditions (Huch et al., 2015) and further extended to extrahepatic/intrahepatic cholangiocyte organoid systems (Sampaziotis et al., 2017; Tysoe et al., 2019; Verstegen et al., 2020; Roos et al., 2022). In parallel, patient-derived organoids have been generated from biliary tract tumors, including cholangiocarcinoma and related BTC entities, providing clinically relevant platforms for modeling and functional testing (Broutier et al., 2017; Saito et al., 2019; Maier et al., 2021; Lee et al., 2023). While these tissue-based platforms have provided a major advance, their broad implementation is still constrained by sample accessibility and variability in tissue quality. In this setting, bile offers a practical alternative starting material that can be obtained during routine clinical procedures and, when processed appropriately, can yield expandable epithelial cultures that are suitable for standardized workflows, while preserving the limited histological tissue available in this tumor type (Soroka et al., 2019a; 2019b; Roos et al., 2021b; Kinoshita et al., 2023).

In line with recent community efforts to harmonize hepatopancreatobiliary organoid definitions and reporting standards (Marsee et al., 2021), we present an operator-oriented, step-by-step protocol for establishing biliary organoids from ERCP bile. Our workflow was developed and tested in a real-world cohort encompassing both benign and malignant strictures (five benign biliary obstructions due to choledocholithiasis and 16 malignant strictures, including 13 CCA, one GBC, and two ampullary tumors). Beyond the standard methods’ descriptions, we explicitly detail the practical steps and decision points that are often under-reported yet can substantially affect the success rates. By formalizing these critical control points, the protocol aims to improve the reproducibility and facilitate implementation of bile as an input material for biliary organoid generation, complementing previously published bile-based protocols (Soroka et al., 2019b). Although ERCP is currently the most common source of bile for these applications and was used here, bile-derived organoids have also been initiated from alternative clinical routes (e.g., gallbladder-derived bile or percutaneous procedures), supporting broader applicability beyond ERCP-only workflows (Roos et al., 2021b).

A relevant consideration specific to bile from malignant strictures is the frequent coexistence of normal-like and tumor-like epithelial populations during early culture, reflecting the biological mixture of shed epithelial cells present in bile (Kinoshita et al., 2023). Although this heterogeneity can complicate interpretation when tumor-specific readouts are required, it can also be leveraged as a unique strength of bile-based sampling as a single clinical specimen may provide access to both malignant and non-malignant organoids from the same individual, thus enabling within-patient comparisons and matched control systems. Practically, when the goal is to maintain both the populations, physical separation by manual handpicking can be used to derive and propagate distinct lines from morphologically different organoids, as described in tumor organoid workflows (Broutier et al., 2017). Conversely, when the experimental goal is only to enrich for tumor-derived organoids, additional strategies may counteract the preferential outgrowth of normal cholangiocyte organoids under routine expansion conditions. In this context, Kinoshita et al. proposed complementary approaches including repeated passaging, xenografting, and selective pressure using an MDM2 inhibitor in TP53 mutation-harboring cases (Kinoshita et al., 2023). In line with this, our pilot targeted sequencing and ULP-WGS analyses in selected paired bile–organoid samples support that organoid-derived DNA can recover alterations detected in bile, although the tumor-associated signals may be reduced compared with the matched bile specimen. Future applications of this workflow may benefit from systematic handpicking-based separation followed by molecular profiling, permitting the generation of paired normal-like and tumor-enriched lines from the same bile specimen for lineage-resolved comparisons.

Finally, to extend bile-derived organoids from descriptive modeling to functional interrogation, we integrate two complementary siRNA delivery workflows: one performed in dissociated organoid cells prior to re-embedding and one performed directly in fully formed 3D organoids. Efficient gene perturbation in 3D cultures remains more variable than in 2D systems due to extracellular matrix barriers and the compact architecture of organoids. Accordingly, workflows frequently implement the delivery of siRNAs at the dissociated/single-cell stage prior to re-embedding (Aloia et al., 2019), whereas knockdown in intact 3D structures is generally more challenging and less standardized (Laperrousaz et al., 2018; Morgan et al., 2018). By detailing handling conditions that preserve organoid integrity while enabling uptake and providing proof-of-concept knockdown validation (siRNA targeting gene X-mRNA in both dissociated and fully formed organoids), this workflow supports functional gene interrogation in a clinically accessible biliary organoid system.

Overall, this protocol expands the practical repertoire of biliary models by enabling reproducible generation of bile-derived organoids across benign and malignant strictures and incorporating functional siRNA perturbation approaches that are suitable for both early and established 3D cultures. We anticipate that the standardized bile-derived organoid workflows will facilitate mechanistic studies in cholangiopathies, enable matched patient-derived comparisons, and support translational pipelines such as biobanking and functional testing.

## Data Availability

The original contributions presented in the study are included in the article/Supplementary Material; further inquiries can be directed to the corresponding authors.
