# Supplementary material for: A guide for establishing patient-derived organoids from bile samples obtained during endoscopic procedures and performing gene expression knockdown
Source: Front Cell Dev Biol. 2026 May 29;14:1812445. doi: 10.3389/fcell.2026.1812445 (PMC13260716; doi:10.3389/fcell.2026.1812445)
Supplement: Supplementary file 1 [file DataSheet1.pdf]

Supplementary Material

A guide for establishing patient-derived organoids from bile samples obtained during endoscopic procedures and performing gene expression knockdown

Carla Rojo[1], Juan J Vila[2,3], Laura Guembe[4], Amaia Arrubla-Gamboa[2,3], Vanesa Jusué-Irurita[2,3], Juan Carrascosa-Gil[2,3], María Rullan[2,3], Javier Randez-Garbayo[2], Maite G Fernández-Barrena[1,3,5], Meritxell Huch[6,7], Jesús Urman[2,3], Matías A Ávila[1,3,5], Carmen Berasain[1,5,*], Maria Arechederra[1,3,5,*].

1 Supplementary Figures

2 Supplementary Tables

Supplementary Figure S1. X mRNA levels in dissociated and fully formed bile-derived organoids at 96 h. qPCR analysis of *X* mRNA levels at 96 h after siRNA-mediated silencing in bile-derived organoids. **(A)** Dissociated organoid workflow. **(B)** Fully formed organoid workflow. In both cases, organoids were transfected with siX or control siRNA (siGL), and *X* mRNA levels were measured by qPCR. Data are shown as relative expression normalized to the corresponding control condition.

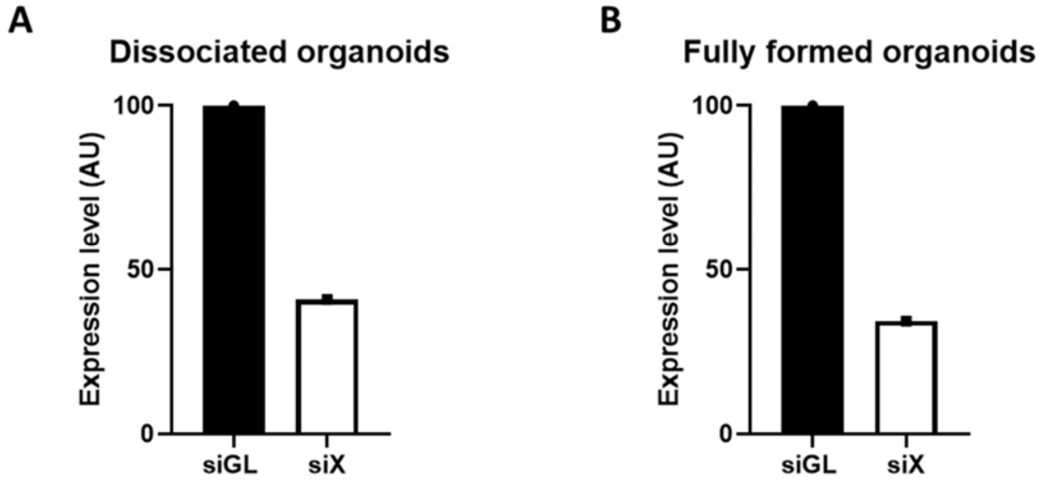

Table S1. Composition of the conditioned culture medium (CCM). The table details the reagents, suppliers, stock concentrations, final working concentrations, and volumes required to prepare 10 mL of complete conditioned medium (CCM). The basal culture medium (BCM) was prepared as described in Section 2.5 and subsequently supplemented with Wnt3A-conditioned medium, growth factors, small-molecule inhibitors, and supplements. All components were added under sterile conditions using the indicated stock solutions. Single-use aliquots were employed to avoid repeated freeze–thaw cycles.

**Reagent**	**Reference**	**Manufacturer**	**Stock concentration**	**Final concentration**	**Volume for 10 mL**
Basal Culture Medium (BCM)	12634-010	Gibco	–	–	4.4 mL
Primocin	ant-pm2	InvivoGen	50 mg/mL	100 µg/mL	20 µL
3dGRO™ L-WNT Conditioned Media	SCM105	Merck	2×	1×	5 mL
N2 supplement	17502048	Life Technologies	100×	1×	100 µL
B27 supplement (w/o vitamin A)	12587010	Life Technologies	50×	1×	200 µL
Nicotinamide	N0636-100G	Merck	1 M	10 mM	100 µL
N-Acetylcysteine	A8199-10G	Sigma-Aldrich	500 mM	1.25 mM	25 µL
Y-27632 (ROCK inhibitor)	Y-27632	Tocris	10 mM	10 µM	10 µL
[Leu15]-Gastrin I human	G9145-0.1MG	Sigma-Aldrich	100 µM	10 nM	1 µL
Recombinant human HGF	100-39H	PeproTech	25 µg/mL	25 ng/mL	10 µL

Recombinant human FGF10	100-26	PeproTech	100 µg/mL	100 ng/mL	10 µL
Recombinant human EGF	AF-100-15	PeproTech	50 µg/mL	50 ng/mL	10 µL
A83-01 (TGF-β inhibitor)	SML0788	Merck	500 µM	5 µM	100 µL
Forskolin	344270	Calbiochem	8 mM	10 µM	12.5 µL

Table S2. Success rate in the recovery of cryopreserved bile-derived organoids. Summary of post-thaw recovery outcomes for cryopreserved bile-derived organoid cultures. For each thawing attempt, the table indicates the patient ID, the time elapsed between cryopreservation and thawing, and whether organoid growth resumed after thawing and re-embedding in Matrigel.

	**Days from cryopreservation to thawing**	**Success thawing rate (%)**
**#1**	14	Yes
	700	No
**#3**	150	Yes
	175	Yes
**#4**	93	Yes
**#5**	27	Yes

Table S3. Practical pitfalls, limitations and troubleshooting across the bile-derived organoid workflow. Summary of practical pitfalls, current limitations and recommended troubleshooting measures across the bile-derived organoid workflow. Items are organized by workflow stage, including organoid establishment, maintenance and expansion, cryopreservation, gene silencing and downstream molecular applications.

Practical limitation / pitfall	Possible cause / rationale	Recommendation / interpretation
**A. Establishment from fresh bile**		
**Reduced establishment success**	Delayed processing, suboptimal transport, or intrinsic sample heterogeneity	Keep bile at 4 °C and process within 2 h. Use sterile additive-free tubes and avoid samples collected after contrast injection.
**No visible pellet after centrifugation**	Low apparent cellular content	Proceed with the protocol, as organoids may still establish. Minimize sample loss during supernatant removal.
**Prominent red pellet / erythrocyte contamination**	Blood contamination during sampling	Perform ACK lysis if needed, but avoid more than two consecutive cycles. Proceed even if a slight red tinge persists.
**Inherent heterogeneity of bile samples**	Variability in volume, cellular content and clinical context	Organoids may still grow even when the initial material appears limited.
**B. Embedding, maintenance and expansion**		
**Poor Matrigel dome integrity**	Matrigel warming, excessive dome volume, or slow handling	Keep Matrigel cold, use pre-chilled plasticware and cut tips, limit dome volume to 35 µL, and pre-warm plates before seeding.
**Cell settling during polymerization**	Delayed or absent plate inversion	Invert the plate immediately after plating and polymerize for 35–40 min at 37 °C.
**Darkening or increased debris**	Overgrowth, nutrient depletion, stress, or delayed passaging	Refresh medium twice weekly and passage organoids before marked darkening. Avoid prolonged enzymatic digestion.
**Poor recovery after passaging**	Incomplete Matrigel disruption or suboptimal digestion	Ensure efficient dome disruption, adapt TrypLE volume to pellet size, and standardize mechanical dissociation.
**Drift toward cystic/non-malignant morphology**	Preferential expansion of non-malignant cholangiocyte-like organoids	Consider manual handpicking or additional enrichment strategies when tumour-enriched cultures are required.
**C. Cryopreservation and thawing**		
**Variable post-thaw recovery**	Differences in organoid condition at freezing, thawing efficiency, or vial-to-vial variability	Freeze organoids during exponential growth (3–5 days post-passaging) and perform rapid thawing.

**Limited evidence on long-term cryostorage**	Post-thaw recovery was assessed in a limited number of cases	Interpret long-term storage performance cautiously; in our hands, successful recovery was observed after up to 175 days of cryopreservation.
**D. Gene silencing workflows**		
**Low silencing efficiency in dissociated organoids**	Cell attachment during incubation may reduce siRNA uptake	Maintain cells in suspension throughout the 5 h incubation and use gentle rocking at 32 °C.
**Variable silencing efficiency between wells**	Uneven cell seeding or inconsistent transfection mix preparation	Prepare a single master cell suspension and master transfection mix for each condition before distributing across wells
**Loss of integrity in fully formed organoids**	Excessive pipetting or mechanical stress	Use cut tips, handle organoids gently, and minimize manipulation outside the incubator.
**Variable knockdown readout**	Differences in timing, transcript/protein kinetics, or line-to-line variability	Use consistent readout time points and confirm silencing by orthogonal methods such as WB and qPCR.
**Limited material for parallel analyses**	Organoid expansion is technically demanding and yields limited material per condition	Prioritize readouts according to the experimental objective and interpret partial validation datasets accordingly.
**E. Downstream molecular applications and general considerations**		
**Low DNA yield or inconsistent quantification**	Sample loss, inhibitors, or measurement bias	Use automated extraction when possible and quantify DNA by Qubit and/or NanoDrop. Interpret purity ratios carefully.
**Discordant variants between bile and matched organoids**	Tumour heterogeneity, sampling differences, or selection during culture	Interpret bile and organoid profiling as complementary readouts and avoid over-interpreting discordance as technical failure.
**Limited cohort size for downstream validation**	Molecular analyses were performed in selected representative cases	Interpret targeted NGS, ULP-WGS and silencing validation as proof-of-concept applications rather than comprehensive benchmarking.
**Contamination**	Non-sterile handling or contaminated reagents	Use strict aseptic technique, test routinely for mycoplasma, and discard contaminated cultures.
